# Yoga, Meditation and Mind-Body Health: Increased BDNF, Cortisol Awakening Response, and Altered Inflammatory Marker Expression after a 3-Month Yoga and Meditation Retreat

**DOI:** 10.3389/fnhum.2017.00315

**Published:** 2017-06-26

**Authors:** B. Rael Cahn, Matthew S. Goodman, Christine T. Peterson, Raj Maturi, Paul J. Mills

**Affiliations:** ^1^Department of Psychiatry and Behavioral Sciences, University of Southern California Los Angeles, CA, United States; ^2^Brain and Creativity Institute, University of Southern California Los Angeles, CA, United States; ^3^California School of Professional Psychology, Alliant International University San Diego, CA, United States; ^4^Center of Excellence for Research and Training in Integrative Health, Department of Family Medicine and Public Health, University of California, San Diego La Jolla, CA, United States; ^5^Midwest Eye Institute Indianapolis, IN, United States; ^6^Department of Ophthalmology, Indiana University School of Medicine Indianapolis, IN, United States; ^7^Chopra Foundation Carlsbad, CA, United States

**Keywords:** yoga, meditation, BDNF, cortisol, inflammatory markers, inflammation, stress

## Abstract

Thirty-eight individuals (mean age: 34.8 years old) participating in a 3-month yoga and meditation retreat were assessed before and after the intervention for psychometric measures, brain derived neurotrophic factor (BDNF), circadian salivary cortisol levels, and pro- and anti-inflammatory cytokines. Participation in the retreat was found to be associated with decreases in self-reported anxiety and depression as well as increases in mindfulness. As hypothesized, increases in the plasma levels of BDNF and increases in the magnitude of the cortisol awakening response (CAR) were also observed. The normalized change in BDNF levels was inversely correlated with BSI-18 anxiety scores at both the pre-retreat (*r* = 0.40, *p* < 0.05) and post-retreat (*r* = 0.52, *p* < 0.005) such that those with greater anxiety scores tended to exhibit smaller pre- to post-retreat increases in plasma BDNF levels. In line with a hypothesized decrease in inflammatory processes resulting from the yoga and meditation practices, we found that the plasma level of the anti-inflammatory cytokine Interleukin-10 was increased and the pro-inflammatory cytokine Interleukin-12 was reduced after the retreat. Contrary to our initial hypotheses, plasma levels of other pro-inflammatory cytokines, including Interferon Gamma (IFN-γ), Tumor Necrosis Factor (TNF-α), Interleukin-1β (IL-1β), Interleukin-6 (IL-6), and Interleukin-8 (IL-8) were increased after the retreat. Given evidence from previous studies of the positive effects of meditative practices on mental fitness, autonomic homeostasis and inflammatory status, we hypothesize that these findings are related to the meditative practices throughout the retreat; however, some of the observed changes may also be related to other aspects of the retreat such as physical exercise-related components of the yoga practice and diet. We hypothesize that the patterns of change observed here reflect mind-body integration and well-being. The increased BDNF levels observed is a potential mediator between meditative practices and brain health, the increased CAR is likely a reflection of increased dynamic physiological arousal, and the relationship of the dual enhancement of pro- and anti-inflammatory cytokine changes to healthy immunologic functioning is discussed.

## Introduction

Decades of research have linked meditation-based practices to positive psychological and health outcomes in both clinical and nonclinical populations (Grossman et al., 2004; Cahn and Polich, 2006; Rubia, 2009; Chiesa and Serretti, 2010; Keng et al., 2011; Davidson and McEwen, 2012). More recently, researchers have sought to understand the biological mechanisms mediating subjective improvements in well-being, including physiological, neuroendocrine, immune and genomic changes. Increasing evidence points towards the possibility that sustained engagement with meditative practices has both salubrious psychological and neurophysiological benefits (Cahn and Polich, 2006; Dahl et al., 2015; Tang et al., 2015; Luders and Cherbuin, 2016) and positively enhances biological functioning through both hypothalamic pituitary adrenal (HPA) effects and the regulation of inflammatory processes (Bower and Irwin, 2015).

Brain-derived neurotrophic factor (BDNF) is a neuroregulatory modulator that has received a paucity of attention in meditation research to date. BDNF is a key neurotrophin that promotes development, survival and plasticity of neurons in the central and peripheral nervous systems (Huang and Reichardt, 2001; Binder and Scharfman, 2004). This potent neuromodulator is most active in brain areas that play pivotal roles in learning, memory and higher cognition such as the hippocampus and cortex. Likely related to its ability to cross the blood-brain barrier, peripheral serum levels of BDNF are correlated with cerebrospinal fluid levels (Pan et al., 1998). In addition, BDNF is involved in the complex regulation of many diverse aspects of physiology such as inflammation, immunity, mood regulation, stress response and metabolism (Papathanassoglou et al., 2015). Decreased BDNF levels have been associated with psychiatric and neurological disorders including anxiety, depression and Alzheimer’s disease (Phillips et al., 1991; Chen et al., 2006; Bocchio-Chiavetto et al., 2010), as well as emotional exhaustion and burnout (Onen Sertoz et al., 2008). We assessed BDNF levels in this study of the psychoneuroimmunologic effects of a sustained yoga and meditation retreat in order to investigate the possibility that BDNF may play a key signaling role in the promotion of stress resilience and integrated mind-body wellness.

The HPA axis is an important neuroendocrine system whose activation underlies the stress response (de Kloet et al., 2005). Accumulating evidence links excessive adrenocortical activity and elevated basal cortisol levels to the progression of psychiatric and other medical illness (Brown et al., 2004; de Kloet et al., 2005; Zannas et al., 2015). Cortisol, a glucocorticoid secreted by the adrenal glands in response to adrenocorticotropic hormone (ACTH) released from anterior pituitary gland, is a reliable marker of HPA activity and follows a robust circadian rhythm, which peaks 30 min after awakening (termed the “Cortisol Awakening Response”—CAR) and gradually declines throughout the day (Akerstedt and Levi, 1978; Wüst et al., 2000). Thus, while basal cortisol levels, typically measured in the late afternoon or evening, are a reliable measure of the relative activation of the sympathetic nervous system, there is also an important and separate relationship between the homeostatic fitness of the autonomic nervous system and robust circadian variations in cortisol levels, with sympathetic hyperactivity demonstrating a tendency to reduce the circadian variability of cortisol levels (Chida and Steptoe, 2009).

Another important and related biological mediator of health and wellness is the inflammatory pathway. Research over the past 20 years has shown that pro-inflammatory bodily states are associated with a propensity towards mental disorders such as depression and anxiety (Miller et al., 2009; Slavich and Irwin, 2014; Furtado and Katzman, 2015; Strawbridge et al., 2015) as well as an array of chronic medical illnesses (Aggarwal et al., 2006; Swardfager et al., 2010). Generally, decreases in inflammatory pathway activation during periods without active infection are associated with better physical and mental well-being (Elenkov et al., 2005); however, acute inflammatory responses are also adaptive, so the general decrease of pro-inflammatory (and increase in anti-inflammatory) immune mediators is not always a clear marker of health and wellness, and instead a healthy homeostatic balance between pro- and anti-inflammatory signaling is most adaptive (Black, 2002).

Chronic inflammatory states can be triggered through psychosocial stress (Steptoe et al., 2007; Miller et al., 2009; Irwin and Cole, 2011), and psychosomatic interventions like meditative practices that invoke stress-reduction mechanisms and psychophysiological self-regulation have demonstrated anti-inflammatory benefits (Bower and Irwin, 2015). Inflammatory states can be measured through circulating pro-inflammatory cytokines such as Interferon Gamma (IFN-γ), Interleukin-1β (IL-1β), Interleukin-6 (IL-6), Interleukin-8 (IL-8), Interleukin-12 (IL-12), and Tumor Necrosis Factor (TNF-α) in addition to anti-inflammatory cytokines such as Interleukin-10 (IL-10). It is important to note that these cytokines are involved in a complicated and multifactorial regulation towards the end of providing optimal support to effectively fighting off molecular “other” targets and microbes under the surveillance of healthy immune functioning while also not promoting inflammation and inadvertent attack of “self” targets. Indeed some of the assayed cytokines have both pro- and anti-inflammatory potential (IL-6) and upregulation of others supports vigorous immunological defense of viruses and are known to be inhibited by stress (IFN-γ). For example although IL-6 is associated with pro-inflammatory pathways when stimulated through psychological stress, fever, or the acute phase response, IL-6 is also a myokine known to have anti-inflammatory effects when stimulated through exercise (Barton, 1997; Tilg et al., 1997; Opal and DePalo, 2000).

Given accumulating evidence that yoga and meditative practices modulate inflammatory and HPA activity (Jevning et al., 1978; Brown and Gerbarg, 2005; Kiecolt-Glaser et al., 2010; Bower and Irwin, 2015) and the possibility that yoga and meditative practices may positively impact BDNF levels as a mechanism towards salubrious neurophysiological functioning, we sought to investigate the effects of yoga and meditation on BDNF as well as the activity of the HPA axis and inflammatory markers. Participants were assessed before and after an intensive yoga and meditation retreat. The mind-body practices in this *Isha* Yoga retreat were directed by the leader of this contemporary Yogic tradition. The yogic practices incorporated physical postures, controlled breathing practices, and seated meditations which include focusing on mantra repetition, breath, emptiness or “no thought” and bodily sensation.

A subset of participants within a large 200 participant 3-month Isha yoga retreat volunteered to donate blood and saliva samples before and after the retreat. Here, we report the effects of the retreat on BDNF, salivary cortisol, inflammatory cytokines (i.e., IL-1β, IL-6, IL-8, IL-12, IFN-γ, and TNF-α), IL-10, and psychological variables which included mindfulness, absorption, depression, anxiety and physical complaints. Finally, we investigated the relationship between psychological improvements and biological changes.

## Materials and Methods

### Participants

Women and men participants were tested at the beginning and at the end of a 3-month retreat that occurred at the *Isha* retreat center in the southern US. A subset of 98 participants (mean age: 34.8 years old) agreed to participate in the acquisition of blood and salivary markers and are reported upon here. Due to limited funding, only a subset of participants’ samples was analyzed—38 of the participant ID’s were chosen randomly with the only precondition that half be male (*n* = 19) and half female (*n* = 19). Of these 38, five participant’s plasma data were not used due to the likelihood of acute illness (e.g., flu) based on highly elevated intake (pre-retreat sample) IL-6 levels >8 pg/ml. Seven participant’s saliva data were not used due to extreme outliers of one or more of the six samples collected. An overlapping total of *N* = 26 subjects had usable pre- and post-blood and salivary samples. This study was carried out in accordance with the recommendations of Quorum Independent Review Board with written informed consent from all subjects. All subjects gave written informed consent in accordance with the Declaration of Helsinki. The protocol was approved by the Quorum Independent Review Board. Participants filled out a standard consent form detailing the procedure involving providing pre and post saliva and blood samples as well as psychometric questionnaires as well as their option to discontinue at any time.

### Participant Yoga and Meditation Background

Prior to the retreat, many participants maintained a frequent yoga and meditation practice, including *Shoonya* meditation (sitting practice focused on a state of “non-doing”), *Samyama* meditation (sitting practice with breath-focused open awareness) as well as diverse *Hatha* yoga (mindful movement/stretching) and *pranayama* (focused breath control) practices. The frequency of such practice prior to beginning the retreat was collected prior to the retreat. As a group, participants reported an average of about 2 h (*M* = 127.5 min, *SD* = 41.22 min) daily practice, and an average of about 4.5 years (*M* = 4.54 years, *SD* = 3.26 years) of regular yoga and meditation practice; see Demographics (Table 1) for details.

**Table 1:** Demographics.

	Mean (SD)	Range
Gender	19 M:19 F	
Age (years)	34.28 (8.84)	21–59
Height (inches)	67.18 (4.20)	60–75
Weight (pounds)	142.26 (30.03)	96.2–216.0
Body Mass Index (BMI) (kg/m^2^)	22.05 (3.70)	17.04–34.38
Years of yoga/Meditation experience	4.54 (3.26)	0.2–15
Length of daily practice (min)	127.50 (41.22)	45–180

### Description of the Retreat Intervention

The 3-month residential retreat involved daily meditation and yoga practices and was accompanied by a vegetarian diet and a full daily schedule; participants did not engage in their normal work activities, but did work on projects around the retreat center. For the first 6 weeks of the retreat all participants practiced “*Samyama*,” a breath-focused open awareness meditation practice for 30–50 min and “*Shoonya*,” a conscious non-doing/“no thought” meditation practice for 30 min daily. They also practiced specific practices involving seated yoga postures some of which incorporated bodily movements, and a focus on mantra and specific bodily sensations (called *kriyas*) for about 1–2 h daily. For the last 6 weeks in place of *Samyama, Shoonya* and *kriya* practices, participants practiced a form of meditation referred to as *Linga sanchalana*, which is a focused attention meditation practice, for approximately 1 h twice daily. Finally, in addition to seated meditation practices, participants also practiced Hatha yoga ~1–2 h daily and chanting ~1 h daily. In sum, participants practiced approximately 2 h of sitting meditation practices, 1–2 h of yoga practice with a meditative component and 1 h of chanting daily.

### Assessment Schedule

During the first week of the retreat, participants reported pre-retreat (time 1; T1) psychometric measures and gave pre-retreat salivary and blood samples. During the last week they reported post-retreat (time 2; T2) psychometric measures and gave post-retreat salivary and blood samples. Specifically, they gave a morning fasting blood sample on retreat day 3, and then again gave a morning fasting blood sample on retreat day 87. On these same days, participants filled out pre-retreat psychometric questionnaires (day 3) and post-retreat psychometric questionnaires (day 87). They also produced six salivary samples at specific time points as described below on retreat day 4, and then again gave six salivary samples at the same time points during the last week of the retreat on retreat day 88.

### Psychometric Scales

Psychometric questionnaires included the Brief Symptom Inventory-18 (BSI-18; Derogatis, 2000), the Freiburg Mindfulness Inventory (Walach et al., 2006), and the Tellegen Absorption Scale (Tellegen and Atkinson, 1974).

### Salivary Cortisol

Diurnal output of cortisol was assessed by having subjects collect saliva as they went about a day of normal retreat activities within the first week of the retreat—day 3—and once again during the last week of the retreat—day 87. To facilitate the collection process, we asked participants to collect the first sample immediately upon awakening (4:45 AM), and then throughout the day at the following time points: 30 min post-awakening (5:15 AM), 9:15 AM during the break in between yoga practices and meditation, 12:30 PM just before lunch, 5:10 PM during a break, and lastly at 8:30 PM after meditation, just prior to bed. Saliva of each participant was collected in their mouth and then passively dropped into a Salimetrics saliva collection tube (Carlsbad, CA, USA). The tubes from T1 at the beginning of the retreat were then placed in a freezer at −20°C. The same procedure was followed at T2 during the last week of the retreat. Two days after the collection of the T1 and T2 saliva samples, the tubes from all subjects were placed on dry ice and sent to the UC San Diego Clinical Research Biomarker Laboratory. Upon arrival to the lab, all saliva samples, were immediately stored at −80°C and saliva cortisol levels were determined by commercial ELISA (Salimetrics, Carlsbad, CA, USA). Intra- and inter-assay coefficients were <8%.

### BDNF and Inflammatory Biomarkers

BDNF and inflammatory biomarker levels were assessed in plasma by obtaining EDTA blood from participants early in the morning after awakening and prior to breakfast, on day 4 (T1) of the 90-day program and again on day 88 (T2) of the 90-day program. For both T1 and T2 samples participants were instructed not to engage in any exercise prior to the blood sample acquisition in the morning. The blood was drawn in 10 cc EDTA (purple top) tubes and the tubes were placed on ice for up to 30 min then spun at 400× *g* for 15 min at 4°C. The supernatant was then aspirated and placed into labeled microcentrifuge tubes (3 cc each) on dry ice immediately. Both T1 and T2 samples were kept on dry ice in labeled trays overnight and sent the next day by overnight delivery to the UC San Diego Clinical Research Biomarker Laboratory for quantitation. Upon arrival to the lab, samples were immediately stored at −80°C. T1 and T2 samples were subjected to analysis simultaneously and in parallel, approximately 2 months after T2 data collection. Plasma levels of BDNF, IFN-γ, TNF-α, IL-1β, IL-6, IL-8, IL-10 and IL-12 were all determined by commercial ELISA kits (R&D Systems, Minneapolis, MN, USA). Intra- and inter-assay coefficients were <5%.

### Statistical Analysis

For the salivary cortisol, a 2 × 6 Repeated measures ANOVA was conducted with factors being (pre- vs. post-retreat) × (6 time points in the day). Greenhouse-Geisser corrections for multiple comparisons were run and Tukey *post hoc* analysis to analyze any significant interactions. For the plasma BDNF and inflammatory biomarker analysis, the plasma values were found to vary significantly from normal distribution, thus values were natural log-transformed to produce more normal distributions. Values greater than three standard deviations from the mean were removed, with the # participants removed ranging from one to three subjects for each of the biomarkers analyzed. For each biomarker, a paired *t*-test for dependent samples was run on the natural log transformed T1 (pre-retreat) and T2 (post-retreat) plasma levels of the biomarkers to assess for significant differences across time from pre- to post-retreat. To assess effects of gender, age, BMI, and change in BMI an ANOVA was also run on the T1 vs. T2 biomarker levels with these demographic categorical/continuous predictors. A limited number of hypothesized correlations between changes in inflammatory biomarkers, BDNF, and cortisol levels and symptoms of psychological distress as measured by the Brief Symptom Inventory were assessed for significance using basic linear correlation procedures. All statistical analyses were run in Statistica 8.0 (Statsoft, Tulsa, OK, USA).

## Results

Paired *t*-test analysis of psychometric data (see Table 2) showed that these healthy normal volunteers scored quite low on the BSI-18 questionnaire (mean scores pre-retreat on the 0–72 scale = 10.5 and post-retreat = 4.1). Nonetheless, there were clearly significant improvements in the Brief Symptom Inventory-18 scores, including the total score (*t*_(33)_ = 4.66, *p* < 0.0001) as well as each of the subscores which included depressive (*t*_(33)_ = 2.84, *p* < 0.01), anxious (*t*_(33)_ = 4.22, *p* < 0.0001), and somatic (*t*_(33)_ = 4.66, *p* < 0.0001) symptoms. In addition, there was an increase in mindfulness as assessed by the Freiburg Mindfulness Inventory (*t*_(33)_ = 4.42, *p* < 0.0001), and no change on absorption as measures by the Tellegen Absorption Scale (*p* = 0.4).

**Table 2:** Psychometrics pre- and post-retreat.

*N* = 34	Pre mean (SD)	Post mean (SD)	*t*	*df*	*p*
BSI-18 Total	10.5 (11.0)	4.12 (6.00)	4.66	33	<0.0001
BSI-Depression	3.09 (4.39)	1.26 (1.96)	2.84	33	<0.01
BSI-Anxiety	3.76 (4.20)	1.21 (2.07)	4.22	33	<0.0001
BSI-Somatic	3.67 (3.62)	1.65 (2.83)	4.66	33	<0.0001
Freiburg mindfulness	39.6 (7.65)	44.5 (7.07)	−4.42	33	<0.0001
Tellegen absorption	88.6 (29.6)	91.3 (28.9)	−0.86	33	0.4

Biological markers analyzed included weight as well as inflammatory biomarkers, BDNF and salivary cortisol. As shown in Table 3, results of paired *t*-tests comparing pre-retreat data to post-retreat data revealed that while the group was on average at a healthy Body Mass Index (BMI) from the outset, small but significant reductions were observed in BMI (*t*_(35)_ = 4.37, *p* < 0.0001). In regards to the plasma biomarker data, paired *t*-tests on the normalized data, after removing extreme outliers, indicated increases in BDNF (*t*_(31)_ = −4.03, *p* < 0.001), IFN-γ (*t*_(31)_ = −2.62, *p* < 0.05), TNF-α (*t*_(30)_ = −6.37, *p* < 0.000001), IL-1β (*t*_(31)_ = −2.39, *p* < 0.05), IL-6 (*t*_(30)_ = −5.13, *p* < 0.0001), IL-8 (*t*_(30)_ = −3.30, *p* < 0.01), and IL-10 (*t*_(29)_ = −4.65, *p* < 0.0001). In contrast, there was a decrease in the level of IL-12 (*t*_(31)_ = 2.37, *p* < 0.05).

**Table 3:** Biomarkers pre- and post-retreat.

	Pre Mean (SD)		Post Mean (SD)		*t*	*df*	*p*
	Raw	Ln	Raw	Ln			
BMI (kg/m^2^)	22.1 (3.7)	-	21.2 (3.1)	-	4.37	35	<0.0001
BDNF (pg/ml)	2513 (1484)	7.65 (0.64)	7039 (5274)	8.44 (1.12)	−4.03	31	<0.001
IFN-γ (pg/ml)	0.88 (0.56)	−0.32 (0.68)	1.11 (0.65)	−0.06 (0.62)	−2.62	31	<0.05
TNF-α (pg/ml)	3.99 (1.06)	1.35 (0.25)	4.59 (0.96)	1.5 (0.2)	−6.37	30	<0.000001
IL-1β (pg/ml)	0.28 (0.12)	−1.37 (0.47)	0.35 (0.23)	−1.19 (0.49)	−2.39	31	<0.05
IL-6 (pg/ml)	0.79 (0.59)	−0.41 (0.58)	1.18 (0.75)	−0.01 (0.61)	−5.13	30	<0.0001
IL-8 (pg/ml)	3.11 (0.85)	1.1 (0.28)	3.62 (0.93)	1.25 (0.26)	−3.30	30	<0.01
IL-10 (pg/ml)	3.46 (4.57)	0.88 (0.72)	4.25 (5.70)	1.07 (0.73)	−4.65	29	<0.0001
IL-12 (pg/ml)	12.05 (41.9)	0.36 (1.5)	12.05 (42.8)	0.23 (1.6)	2.36	31	<0.05

**Figure 1:**
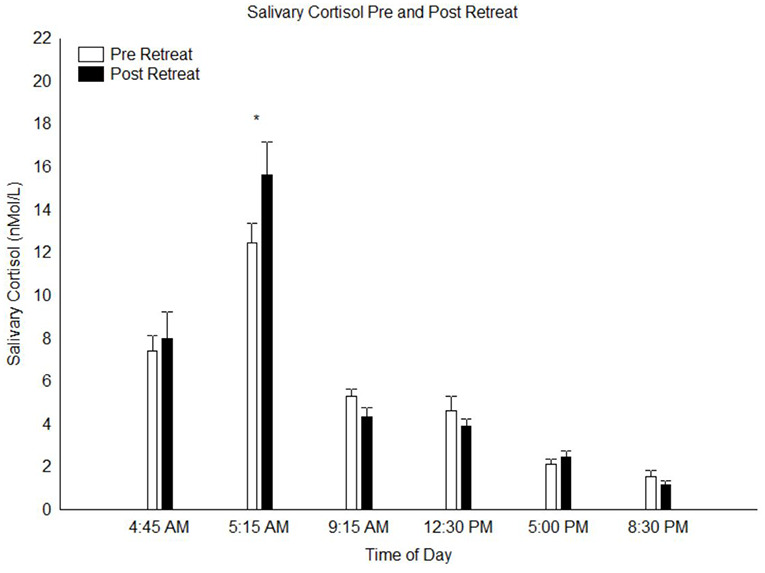
Salivary cortisol levels throughout the day at pre- and post-retreat, *N* = 31. *p < 0.05.

A calculation of the area under the curve of the salivary cortisol concentration across the day was done and a paired *t*-test of this data indicated no difference between pre and post retreat samples (*p* = 0.3). A repeated measures 2 × 6 ANOVA conducted on the salivary cortisol measures with factors “pre vs. post retreat” and “time of day” yielded no main effect of pre vs. post retreat (*p* = 0.50) but a highly significant main effect time of day (*F*_(5,150)_ = 89.8, *p* < 0.0000001), indicating the pronounced circadian variation of cortisol levels across the day. There was also a significant interaction between pre vs. post retreat and time of day (*F*_(5,150)_ = 3.31, *p* < 0.01). *Post hoc* analysis of this significant interaction indicated that at T2, cortisol levels 30 min post-awakening (peak cortisol level associated with the CAR) was significantly higher in the post-retreat compared to the pre-retreat samples (*p* < 0.01; see Figure 1). At all other time points throughout the day from awakening to bed time, there was no significant difference in the pre vs. post retreat cortisol levels (all *p* > 0.9).

Including BMI, change in BMI, age and gender as covariates in the ANOVA of the salivary cortisol data (gender as categorical predictor, age and BMI characteristics as continuous predictors), revealed no significant interaction between these demographic characteristics and the significant effect on the salivary CAR. There was, however, a significant main effect of gender on salivary cortisol levels such that women showed higher overall cortisol levels across the day than men (*p* = 0.05). In addition, these demographic characteristics were entered as predictors into the ANOVA analyses of the effects on the plasma inflammatory biomarkers and BDNF levels. Here we found significant interactions only between gender and three of the dependent variables: BDNF, IFN-γ and IL-10. For BDNF there was a main effect of gender such that women were found to demonstrate higher levels of plasma BDNF than men (*F*_(1,30)_ = 6.88, *p* < 0.05) and there was a nearly significant gender × pre-post interaction (*p* = 0.06) indicating a tendency for women to show greater relative increases in BDNF over the course of the retreat. For IFN-γ a significant gender × pre-post interaction was obtained (*F*_(1,30)_ = 4.33, *p* < 0.05) and *post hoc* analysis indicated that the increases in IFN-γ levels were significant for women (*p* < 0.01) but not men (*p* = 1). For IL-10 a significant gender × pre-post interaction was obtained (*F*_(1,28)_ = 4.90, *p* < 0.05) and *post hoc* analysis indicated that the increases in IL-10 levels were significant for women (*p* < 0.001) but not men (*p* = 0.31). There was no significant interaction between participant age, gender, or BMI and change in the other biomarker levels.

To explore the possible relationship between the changes in biomarker levels among each other and related to psychological changes, a small number of Pearson product-moment correlations between inflammatory markers, CAR dynamics, BDNF plasma levels and psychological measures were executed. The normalized change in BDNF plasma level (natural log of BDNF post-retreat minus natural log of BDNF pre-retreat) was inversely correlated with BSI-18 anxiety scores at both the pre-retreat (*r* = 0.40, *p* < 0.05) and post-retreat (*r* = 0.52, *p* < 0.005) such that those with greater anxiety scores tended to evidence a smaller pre- to post-retreat increase in plasma BDNF levels. No such correlations were observed between other BSI-18 measures or mindfulness scores and BDNF levels and no correlations were obtained between these psychometric measures and CAR findings. A relationship was found between the magnitude of the pre-retreat CAR and the resultant change in BDNF levels, however this relationship was found to be driven by one particular outlier so this relationship was taken to be non-significant. Lastly, in order to assess the unexpected effect demonstrating increases in both the prototypical anti-inflammatory marker IL-10 and the range of pro-inflammatory markers, correlations were run between IL-10 levels and the other interleukins. We found a positive correlation between the change in IL-10 levels and the change in IL-12 levels from pre- to post-retreat, signifying that the participants who demonstrated greater increases in anti-inflammatory IL-10 levels tended to also demonstrate greater increases in pro-inflammatory IL-12 levels.

## Discussion

The results of this 3-month pre-post study indicate that participation in this yoga and meditation retreat was associated with alterations in psychological functioning, neurotrophic pathways, HPA axis activity, and inflammatory pathway signaling suggesting enhanced stress resilience and well-being. As a group, participants showed significant improvements in depression, anxiety, somatic complaints, increases in mindfulness scores, and a small but significant decrease in BMI. A significant 3-fold increase in BDNF plasma level was observed and the change in BDNF plasma levels from pre-retreat to post-retreat was inversely correlated with participants’ self-reported anxiety levels on the BSI-18, suggesting that this increase in BDNF may be related to psychological markers of wellness. In addition, a significant increase in the CAR was observed from pre- to post-retreat, suggesting improvements in the dynamic rhythmicity of adrenocortical activity, a marker of stress resilience. Lastly, significant increases in both pro- and anti-inflammatory biomarkers were observed in an unexpected pattern not seen in previous studies to our knowledge. Overall we hypothesize that the observed pattern of changes provides evidence for enhanced fitness as assessed along the spectrum of levels of psychoneuroendocrine and immunologic assessment.

A highly significant 3-fold increase in plasma BDNF level was observed comparing pre- to post-retreat assessment data. BDNF is a well-described neurotrophic factor of the neurotrophin gene family that acts on neurons of the central and peripheral nervous system, supports the survival of existing neurons, and encourages the growth and differentiation of new neurons and synapses primarily through its action at the TrkB receptor of neurons (Acheson et al., 1995; Huang and Reichardt, 2001). BDNF signaling has been implicated as a mediator of the positive impact of both physical exercise and cognitive engagement on brain fitness and enhanced neurogenesis, learning, and memory (Huang et al., 2014) as well as anti-depressant effects (Russo-Neustadt et al., 2000; Warner-Schmidt and Duman, 2006; Sen et al., 2008). We hypothesize that the increase in circulating levels of BDNF observed here is likely related to improvements in brain fitness and psychological well-being brought about by participation in the yoga and meditation retreat although ruling out a possible contribution from the limited physical exercise components (hatha yoga practices) of the retreat is not possible.

BDNF has been relatively unexplored in work exploring the impact of meditative practices on biology to date, but one previous study showed that meditative yoga practice was related to increases in BDNF as well as heart rate variability (Pal et al., 2014). A second recent study demonstrated a two-fold increase in BDNF when comparing pre- to post-values for a 12-week yoga and meditation lifestyle intervention involving approximately 90 min of practice daily (Tolahunase et al., 2017). In addition, Naveen et al. (2013) demonstrated that both yoga and antidepressant interventions led to increased levels of BDNF in patients with a history of depression. In this study, those patients receiving only yoga had greater improvements in depression, and there was a significant positive correlation between improvements in depression and increases in BDNF specifically in the yoga-only group suggesting that BDNF may play a key role in the antidepressant effects of yogic practice. In contrast, one previous RCT of a hatha yoga intervention in patients with schizophrenia found no changes in BDNF or psychometric measures of resilience levels (Ikai et al., 2014), possibly indicating that such interventions are not as efficacious for individuals with severe psychotic illness, or at least might need to be further adjusted in order to better support wellness in this population.

In the current study, we found that those scoring higher on the BSI anxiety scale (both at pre- and post-testing) tended to demonstrate a less robust increase in BDNF levels, consistent with the notion that BDNF levels were indeed related to psychological wellness. This fits with evidence from others suggesting that BDNF is negatively correlated with depression (Sen et al., 2008; Polyakova et al., 2015) as well as emotional exhaustion and depersonalization (Onen Sertoz et al., 2008) and increases in association with successful treatment of depression (Polyakova et al., 2015).

Extensive previous work has demonstrated increased circulating BDNF levels due to both acute and chronic aerobic exercise regimes (Brunelli et al., 2012; Dinoff et al., 2016). Although physical exercise levels were not specifically controlled for in this study, they were not a prominent component of the retreat and most participants reported being engaged in an approximately equivalent Hatha yoga/exercise routine prior to the retreat. Given that there was some decrease in BMI for participants, we ran a correlation analysis between change in BMI and change in BDNF to explore whether potential exercise-related decreases in body weight might be correlated with BDNF changes and found a complete lack of association with a *r* value of ~0.05.

There are several alternative mechanistic explanations for the increase in BDNF levels observed in this study involving the impact of the meditative components of the yoga and meditation practices. First, given the significant and growing evidence for the highly active attentional engagement and associated robust modulations of frontal brain circuitry supporting the act of meditative practice (Tang and Posner, 2009; Brewer et al., 2011; Lutz et al., 2015; Tang et al., 2015), it is quite possible that the meditative components of this intensive retreat engaged neuroplastic brain mechanisms in a manner analogous to intensive learning regimes which have been shown to promote neuroplasticity partly through increase in BDNF signaling (Korol et al., 2013; Novkovic et al., 2015; Aarse et al., 2016). A second possible mechanism involves the effects of these practices on the autonomic nervous system. Specifically, deep breathing, yoga and meditation practices have been found to increase vagal tone (Brown and Gerbarg, 2005; Khattab et al., 2007) which could be a potential mediating mechanism in the increased BDNF levels. Parasympathetic activation through the vagus nerve has been shown to increase BDNF levels as well as downstream activation of the BDNF receptor TrkB (Follesa et al., 2007; Furmaga et al., 2012). Lastly, given the clear relationship between increased stress and decreases in BDNF signaling (Duman and Monteggia, 2006; Schmidt and Duman, 2007), other central mechanisms related to the impact of meditative practice on decreasing stressful responding to daily life stressors may play an important role in the impact of yoga and meditation on BDNF levels.

Related to the increase in BDNF levels observed here, the hippocampus is an active site of neuroplasticity and contains in the dentate gyrus one of two primary zones of neurogenesis throughout the adult human lifespan. Physical exercise and environment learning have been clearly demonstrated to exert hippocampal plasticity through upregulating BDNF signaling (Vivar et al., 2013; Voss et al., 2013). If the BDNF increases observed here are indeed largely related to the meditative practice component of the retreat, such meditation-related BDNF signaling may be a partial explanation for the repeated findings of increased hippocampal volumes due to meditative practice in both short and long term studies (Hölzel et al., 2008, 2011; Luders et al., 2009; Gotink et al., 2016).

Another possible contributor to the effects observed here on BDNF (and other assessed biomarker) levels is diet, which has a profound impact on the gut microbiota community and resultant microbiome signaling processes within the brain and body. Diet was controlled during the retreat in that participants were fed high-quality vegetarian food prepared by the staff. Studies in mice have demonstrated associations between the gut microbiota and BDNF in the regulation of brain physiology, behavior, and memory (Diaz Heijtz et al., 2011). Thus, the enhanced BDNF expression observed in our participants may be related to a number of mediating mechanisms called into play by the multi-modal yoga and meditation retreat intervention, including attentional and cognitive stimulatory factors brought about by the specific meditative practices as well as the physical exercise component, possible regulations in vagal tone and also diet and other attendant factors to the retreat setting. These factors include the retreat setting in nature, decreased demands of everyday working life, new daily rhythms and social/relational demands called into play by the nature of the retreat setting and activities.

Lastly, it is important to note that including the covariate gender into the effects of the yoga-meditation retreat effects on BDNF revealed an overall gender effect such that BDNF levels in women in this sample were higher than those in men. There was also a nearly significant interaction between gender and the increases in BDNF from pre- to post-retreat such that women tended to demonstrate more dramatic increases in BDNF levels than men. Given that previous work demonstrating generally equivalent plasma BDNF levels in men and women (Lommatzsch et al., 2005), we take the finding of greater BDNF levels in women than men of this study to be related to non-random sampling of the general population. Nonetheless, the near-significance of the gender × time interaction observed here implies the possibility that the effects of this intervention might be mediated through effects on BDNF-related signaling in women more so than men.

The CAR represents the acute early-morning increase in cortisol that functions to enhance alertness, physiological arousal, and preparedness to meet daily demands. The increased CAR we report here is likely related to increases in morning wakefulness and stress resilience (Fries et al., 2009; Clow et al., 2010a,b). These results are consistent with others who have reported increases in the CAR after mindfulness-based practices (Matousek et al., 2011). It is also possible that this intervention improved sleep quality and this, in turn, impacted a more dynamic and robust CAR. In any case, we did not find any significant differences in other aspects of the diurnal or overall cortisol levels from pre- to post-retreat. While some have reported an overall decrease in daily cortisol secretion following mindfulness practice (Carlson et al., 2003, 2007; Witek-Janusek et al., 2008), others have found no change (Robinson et al., 2003; Galantino et al., 2005; Klatt et al., 2009). Matousek et al. (2010) reasonably suggest that such discrepancies might be attributed to methodological differences, such as sample size and population characteristics. In our case, the healthy and seasoned meditators participating in this retreat reported low levels of anxiety and depression symptoms on the BSI-18 from the outset and likely had relatively healthy baseline cortisol levels, leading to a floor effect with regards to detecting potential decreases in cortisol output from wellness-promoting activities. The demonstration of increased CAR in this group suggests that this measure of dynamic cortisol levels across the day/night cycle may be a more sensitive marker of increased physiological stress resilience than absolute cortisol levels in healthy subjects.

An intriguing link between the effects on BDNF and the CAR may be hippocampal functional integrity. As mentioned, increased BDNF levels due to physical exercise has previously been shown to relate with hippocampal neurogenesis and likely relate to its positive effects on well-being and depression. It is reasonable to hypothesize that the increased BDNF levels secondary to this yoga-meditation retreat may relate to positive effects on hippocampal functioning although this was not directly assessed. Interestingly, previous work has shown that hippocampal functioning also serves as a promoting factor for the robust functioning of the CAR (Fries et al., 2009; Clow et al., 2010b). Specifically, clinical patients with hippocampal damage have been shown to evidence greatly reduced CAR dynamics (Buchanan et al., 2004) and positive associations between hippocampal volume and CAR amplitude have been demonstrated (Pruessner et al., 2007). Thus, it may be the case that the wellness and resilience-promoting benefits of yoga and meditation may work through a mechanism involving BDNF upregulation, hippocampal flourishing, and a related boost in CAR amplitude. Further studies aimed at explicating these relationships may reveal important mechanistic explanations for integrated mind-body wellness promotion.

Turning attention to the inflammatory markers assayed here, a statistically significant increase was observed in the anti-inflammatory IL-10 levels, while a significant decrease in pro-inflammatory IL-12 was observed, which supported our hypothesis that the yoga and meditation retreat would impact the inflammatory pathway by reducing tendencies towards inflammatory states. We also observed significant increases in levels of the pro-inflammatory markers TNF-α, IFN-γ, IL-1β, IL-6, and IL-8.

Some of these cytokines, such as IL-6, can induce both pro- and anti-inflammatory effects thus adding an additional layer of complexity (Scheller et al., 2011). In addition, pro- and anti-inflammatory response modulations may be adaptive depending on the context (i.e., baseline states of over vs. underactive inflammatory processing, acute vs. prolonged effects, local vs. systemic responses). Of note, a generally observed cytokine response to stress and mediated through glucocorticoids includes a reduction of the T helper 1 cell (Th1)-mediated production of pro-inflammatory IFN-γ, TNF-α and IL-12 and an activation of T helper 2 cell (Th2)-mediated production of IL-10. Later effects of stress can include induction of IL-1β, IL-8 and TNF-α (Elenkov et al., 2005; Calcagni and Elenkov, 2006). Here we found the unusual pattern of increases in both anti-inflammatory IL-10 in addition to pro-inflammatory TNF-α, IFN-γ, IL-1β, IL-6, IL-8 with simultaneous decreases of IL-12.

To our knowledge, our study is the first to examine a broad range of pro- and anti-inflammatory markers in a healthy population before and after a yoga-meditation intervention, and suggests that for these individuals, intensive meditation and yoga practice can elicit increases in both anti-inflammatory IL-10 levels and concomitant increases in pro-inflammatory TNF-α, IFN-γ, IL-1β, IL-6 and IL-8 levels. This was especially unexpected given the known effect that the prototypical anti-inflammatory cytokine IL-10, produced by Th2 cells, has been shown to result in the inhibition in the synthesis of pro-inflammatory cytokines generally (Mosser and Zhang, 2008), including TNFα, IL-1β, IL-12 and IFNγ (Zheng et al., 1996; Aste-Amezaga et al., 1998; Varma et al., 2001). Explaining this unexpected rather broad increase in both anti- (IL-10) and pro-inflammatory cytokines (with the exception of IL-12) is not trivial and we are not confident in an encompassing explanatory rubric but hypothesize that the pattern may relate to immunologic readiness as explained below.

A meta-analysis of randomized controlled trials of mind-body therapies including yoga, meditation and tai-chi revealed small but not statistically significant decreases in IL-6 and TNF-α following such interventions (Morgan et al., 2014), and a later descriptive review of this literature highlighted the inconsistent nature of the findings with the majority of studies assaying IL-6 and TNF-α demonstrating null effects (Bower and Irwin, 2015). It is important to note inconsistencies in pro- and anti-inflammatory outcomes as well as inconsistent results across studies that have been observed across previous yoga and meditation-based studies. For example, Pullen et al. (2008) showed decreases in IL-6, while Creswell et al. (2012) reported no change in IL-6; Rosenkranz et al. (2013) showed that more meditation practice was associated with lower TNF-α, while Black et al. (2013) reported an increase in TNF-α mRNA expression after yogic meditation; and Witek-Janusek et al. (2008) reported increases in IFN-γ activity in breast cancer patients, while Carlson et al. (2003) reported a decrease in a similar population. These studies tended to differ with respect to the type of intervention (e.g., Kundalini yoga vs. MBSR vs. Tai Chi), population (e.g., clinical vs. non-clinical), setting, design and other methodological factors—lending to the complexities involved in interpreting cytokine and other biomarker samples. In addition, unexpected inflammatory marker expression patterns may be optimally adaptive for different populations. For example, Carlson et al. (2003) and Witek-Janusek et al. (2008) both reported decreases in the anti-inflammatory IL-10 in breast and prostate cancer patients following MBSR, which might reflect a healthy improvement given baseline stress-induced immune dysregulation in this population (Witek-Janusek et al., 2007). Similarly, Yeh et al. (2009) reported an increase in the pro-inflammatory cytokine IL-12 in diabetes patients after Tai chi practice, which was interpreted as related to an improvement toward metabolic regulation.

IL-10, as a prototypical anti-inflammatory cytokine produced by Th2 cells, has been shown capable of decreasing the expression of the Th1-produced pro-inflammatory cytokines. We hypothesize that the increase in IL-10 found here is related to enhanced stress resilience as it is likely to act in concert with the observed increases in pro-inflammatory markers to ensure that inflammatory cascades are not actually being turned on in these subjects. In support of the notion that enhanced resilience is conferred through elevated IL-10 levels is the recent finding assessing inflammatory markers in individuals exposed to urban violence in Brazil. Comparing those individuals who developed PTSD vs. those that did not indicated no difference between groups in IL-6 levels but significantly higher IL-10 levels in the more resilient individuals exposed to trauma but not experiencing PTSD (Teche et al., 2017).

In general, the optimal levels of cytokines are dynamic and related to a fine tuned balance between readiness to engage the inflammatory response when needed and a restraint on excessive inflammation or in the extreme auto-inflammatory states. It is likely that in chronically inflamed body states decreases in pro-inflammatory markers and increases in anti-inflammatory markers is adaptive, but the situation is much less clear in non-inflamed healthy normals. For example, a yoga intervention in breast cancer survivors reported decreases in IL-6, TNF-α, and IL-1β (Kiecolt-Glaser et al., 2010). Breast cancer survivors carry heavy inflammatory burden and thus the yoga intervention may have a more significant anti-inflammatory effect in populations with inflammatory burdens. Similarly, IL-6 and C-reactive protein were reduced following *Hatha* yoga intervention in heart failure patients, which represent another clinical population with a high baseline inflammatory burden, compared to controls (Pullen et al., 2008, 2010). In contrast, our participants scored low on anxiety and depression symptomatology, and when compared to previous literature in healthy normal populations (Kleiner et al., 2013; Krishnan et al., 2014) our levels were decreased in amplitude across the various cytokines obtained, likely indicating that in this sample there was a reduced baseline level of inflammatory marker expression.

Of note, surveying the previous literature we were not able to find previous studies assaying cytokine levels in an equally intensive long-term yoga-meditation retreat. We propose that in relatively healthy adults, intense yogic and meditative practices as assayed in the current study may recruit an integrate brain-body response leading to increases in both pro- and anti-inflammatory signaling processes in support of an upregulated vigorous immunological surveillance system but with concomitant high expression of the anti-inflammatory “break” that IL-10 may represent. Further support of this hypothesis is that despite the known inhibitory effect that IL-10 levels can have on IL-12 expression, we found increased IL-10 levels tended to correlate with increased IL-12 levels in our cohort, providing further indirect support for the hypothesis that an unusual state of regulatory shifts underlies the effects observed in this dataset. Moreover, while we did assess many cytokines involved in inflammatory signaling, these are just the tip of an iceberg of complicated multifactorial signaling pathways regulating this important biological function. It is possible that further studies of a range of pro- and anti-inflammatory cytokines in normal relatively unstressed subjects undergoing salubrious mind-body interventions may replicate our findings of a mixed upregulation of both anti-inflammatory and pro-inflammatory cytokines in which case new theoretical models of broad upregulation of these pathways will need to be formulated.

As with the effect on BDNF described above, another possible explanation for the post-retreat changes in cytokines may be attributed to the physical exercise-related effects of Hatha yoga. For example, acute exercise is known to increase the expression of both pro-inflammatory IL-1β and TNF-α as well as anti-inflammatory IL-6 and IL-10 (Northoff et al., 1994; Ostrowski et al., 1999). Also, relationships between gender and two of the assayed cytokines were obtained, indicating that in this sample the effect of the retreat on increased IFN-γ and IL-10 was driven largely by women in the sample. We were not able to find evidence for pronounced gender-related differences between these cytokines in the literature and have no framework for explaining this gender difference, however it does seem to imply that there are sex-specific mechanisms at play in the response of the immune system to yoga-meditation interventions.

There may be a relationship between the effects we found on BDNF and those with inflammatory markers. Previous studies have demonstrated both anti-inflammatory and pro-inflammatory effects of BDNF—for example, BDNF upregulated IL-10 and transcription factor NF-κB in models of neuroinflammation investigating the neuroprotective effects of BDNF (Jiang et al., 2011) but also increased IL-4 and IFN-γ in *in vitro* studies of human peripheral blood mononuclear cells (Bayas et al., 2003). Moreover, a large body of evidence has demonstrated that the negative impact of inflammation on neurogenesis might be triggered through pro-inflammatory cytokine-induced reductions in BDNF expression/signaling (Calabrese et al., 2014). Here we do not see evidence that pro-inflammatory cytokines decreased BDNF signaling—instead we see very robust increases in BDNF levels as well as increases in some pro-inflammatory cytokines, potentially arguing further for our hypothesis that the observed cytokine changes are related to a system-wide increase in readiness for immunologic challenge.

Lastly, an alternative possible explanation for the observed increase in pro-inflammatory cytokines is that they may have increased because of an aspect of psychological stress inherent in the intensive 3-month yoga-meditation retreat that we did not ascertain using the current design. There may have been some component of increased stress related to the intensive group retreat process with limited free time for independent relaxation and/or the social dynamics related to group practice led by a charismatic teacher as well as being away from familiar and normal activities for a prolonged period. The fact that participants reported relatively little anxiety and depression symptoms at baseline and improvements in these variables argues against this interpretation, as does the increase in BDNF and CAR indicative of improved psychophysiological wellness, but it must be considered.

In sum, the yoga and meditative practices in the 3-month intensive retreat appear to positively impact BDNF signaling, CAR, and immunological markers as well as improve subjective well-being. It is likely that at least some of the significant improvements in both HPA axis functioning as exemplified by the CAR as well as neuroimmunologic functioning as exemplified by increases in BDNF levels and alterations in cytokines were due to the intensive meditation practice involved in this retreat. We hypothesize that the pattern of biological findings is related to enhanced resilience and well-being with increased BDNF signaling possibly related to enhanced neurogenesis and/or neuroplasticity, increased CAR likely related to enhanced alertness and readiness for mind-body engagement, and increased anti- and pro-inflammatory cytokines possibly indicating enhanced immunological readiness. These findings justify further studies of such retreats assessing for the replicability, specificity and long-term implications of these findings. Further research should seek to employ appropriate control groups involved in similar retreat-like interventions without the variable of meditative practices (e.g., relaxation control). In addition, further controls would allow for understanding of the role of other contextual factors (e.g., social dynamics, diet, natural environment, relative impact of a revered spiritual teacher, etc.) affecting the expression and regulation of these biological processes. Future research that assesses broad panels of biomarkers and global studies with more appropriate control populations are needed to clarify the extent to which these changes are related specifically to the meditation and yoga practices, and given the suggestive findings reported here such further work is warranted.

## Author Contributions

BRC: designed study, analyzed results and wrote the manuscript. MSG: analyzed results and wrote the manuscript. RM: organized data collection and analyzed results. CTP: wrote the manuscript. PJM: designed study, organized data quantitation and edited the manuscript.

## Conflict of Interest Statement

This study received funding from co-author RM. RM has no financial relationship with Isha Yoga and had the following involvement with the study: Organized data collection, analyzed results. The corresponding BC and senior PM authors had full executive control over and ownership of the data, processing of biological samples, final analysis, and interpretation of results, as well as the write-up and decision to publish. PM is the Scientific Director of the Chopra Foundation. The Chopra Foundation is a 501 (c) (3) organization dedicated to improving health and well-being partly through research on yoga and meditation and is connected with the Chopra Center that offers meditation and yoga courses.
