# Corrigendum: Yoga, Meditation and Mind-Body Health: Increased BDNF, Cortisol Awakening Response, and Altered Inflammatory Marker Expression After a 3-Month Yoga and Meditation Retreat

**DOI:** 10.3389/fnhum.2022.868021

**Published:** 2022-04-08

**Authors:** B. Rael Cahn, Matthew S. Goodman, Christine T. Peterson, Raj Maturi, Paul J. Mills

**Affiliations:** ^1^Department of Psychiatry and Behavioral Sciences, University of Southern California, Los Angeles, CA, United States; ^2^Brain and Creativity Institute, University of Southern California, Los Angeles, CA, United States; ^3^California School of Professional Psychology, Alliant International University, San Diego, CA, United States; ^4^Center of Excellence for Research and Training in Integrative Health, Department of Family Medicine and Public Health, University of California, San Diego, La Jolla, CA, United States; ^5^Department of Ayurveda and Yoga Research, Chopra Foundation, Carlsbad, CA, United States; ^6^Midwest Eye Institute, Indianapolis, IN, United States; ^7^Department of Ophthalmology, Indiana University School of Medicine, Indianapolis, IN, United States

**Keywords:** yoga, meditation, BDNF, cortisol, inflammatory markers, inflammation, stress

There is an error in the Funding statement. The correct Name for the Funder is Raj Maturi (RM).

The new funding statement should read:

The experimental costs for this work was funded by RM *via* a private personal donation and the authors contributed their time to accomplish the work.

In the original article, there was a previously undisclosed conflict of interest. The corrected conflict of interest statement can be found below:

This study received funding from co-author RM. RM has no financial relationship with Isha Yoga and had the following involvement with the study: Organized data collection, analyzed results. The corresponding BC and senior PM authors had full executive control over and ownership of the data, processing of biological samples, final analysis, and interpretation of results, as well as the write-up and decision to publish. CP is a postdoctoral fellow at the University of California, San Diego partially funded by the Chopra Foundation. PM is the Scientific Director of the Chopra Foundation. The Chopra Foundation is a 501 (c) (3) organization dedicated to improving health and well-being partly through research on yoga and meditation and is connected with the Chopra Center that offers meditation and yoga courses.

The authors apologize for this error and state that this does not change the scientific conclusions of the article in any way. The original article has been updated.

## Publisher's Note

All claims expressed in this article are solely those of the authors and do not necessarily represent those of their affiliated organizations, or those of the publisher, the editors and the reviewers. Any product that may be evaluated in this article, or claim that may be made by its manufacturer, is not guaranteed or endorsed by the publisher.

