# Erratum: Yoga, meditation and mind-body health: increased BDNF, cortisol awakening response, and altered inflammatory marker expression after a 3-month yoga and meditation retreat

**DOI:** 10.3389/fnhum.2023.1278043

**Published:** 2023-08-30

**Authors:** Frontiers Production Office

**Affiliations:** Frontiers Media SA, Lausanne, Switzerland

**Keywords:** yoga, meditation, BDNF, cortisol, inflammatory markers, inflammation, stress

In 2022, the authors submitted a correction in relation to their conflict of interest statement. This needs to be amended on the advice of the Editorial Office.

The original conflict of interest statement read:

“The authors declare that the research was conducted in the absence of any commercial or financial relationships that could be construed as a potential conflict of interest.”

The corrected conflict of interest statement read:

“This study received funding from co-author RM. RM has no financial relationship with Isha Yoga and had the following involvement with the study: organized data collection and analyzed results. The corresponding BC and senior PM authors had full executive control over and ownership of the data, processing of biological samples, final analysis, and interpretation of results, as well as the write-up and decision to publish. CP is a postdoctoral fellow at the University of California, San Diego partially funded by the Chopra Foundation. PM is the Scientific Director of the Chopra Foundation. The Chopra Foundation is a 501 (c) (3) organization dedicated to improving health and well-being partly through research on yoga and meditation and is connected with the Chopra Center that offers meditation and yoga courses.”

The conflict of interest statement now reads:

“This study received funding from co-author RM. RM has no financial relationship with Isha Yoga and had the following involvement with the study: Organized data collection, analyzed results. The corresponding BC and senior PM authors had full executive control over and ownership of the data, processing of biological samples, final analysis, and interpretation of results, as well as the write-up and decision to publish. PM is the Scientific Director of the Chopra Foundation. The Chopra Foundation is a 501 (c) (3) organization dedicated to improving health and well-being partly through research on yoga and meditation and is connected with the Chopra Center that offers meditation and yoga courses.”

In the published article, there was an error regarding the affiliation(s) for Paul J. Mills. As well as having affiliation [4], they should also have [Chopra Foundation, Carlsbad, CA, United States].

In the published article, there was an error regarding the affiliation(s) for Christine T. Peterson. Instead of [4, 5], it should be [4].

The corrected affiliations and author group:

“B. Rael Cahn^1, 2^, Matthew S. Goodman^3^, Christine T. Peterson^4^, Raj Maturi^5, 6^ and Paul J. Mills^4, 7^

^1^ Department of Psychiatry and Behavioral Sciences, University of Southern California, Los Angeles, CA, United States

^2^ Brain and Creativity Institute, University of Southern California, Los Angeles, CA, United States

^3^ California School of Professional Psychology, Alliant International University, San Diego, CA, United States

^4^ Center of Excellence for Research and Training in Integrative Health, Department of Family Medicine and Public Health, University of California, San Diego, La Jolla, CA, United States

^5^ Midwest Eye Institute, Indianapolis, IN, United States

^6^ Department of Ophthalmology, Indiana University School of Medicine, Indianapolis, IN, United States

^7^ Chopra Foundation, Carlsbad, CA, United States”

The publisher apologizes for this mistake. The original article has been updated.

